# Region-associated microbial signatures persist during inoculated fermentation of Nebbiolo grapes

**DOI:** 10.1128/aem.01510-26

**Published:** 2026-09-09

**Authors:** Francesca Cristetti, Paola Di Gianvito, Vasileios Englezos, Gabriele Serafino, Ciro Orecchio, Marco Vincenti, Ilario Ferrocino, Susana Río Segade, Simone Giacosa, Irene Franciosa, Luca Rolle, Luca Cocolin, Kalliopi Rantsiou

**Affiliations:** 1 Department of Agricultural, Forest and Food Sciences, University of Turin 165514 , Grugliasco, Italy; 2 Interdepartmental Centre for Grapevines and Wine Sciences, University of Turin 9314 , Alba, Italy; 3 Department of Chemistry, University of Turin 165514 , Torino, Italy; University of Milano-Bicocca, Milan, Italy

**Keywords:** microbial communities, grape microbiota, *Vitis vinifera *cv. Nebbiolo, fermentation dynamics, volatilome, regional identity

## Abstract

Wine fermentation is driven by dynamic microbial communities that influence fermentation kinetics and wine composition. During fermentation, both the dominant *Saccharomyces cerevisiae* and diverse non-*Saccharomyces* yeasts, along with bacteria, actively contribute to metabolite and aroma formation, potentially reflecting vineyard environmental and geographic characteristics. Despite growing interest in the spatial structuring of grape-associated microbiota, knowledge remains limited regarding the persistence of these microbial signatures during inoculated fermentations. This study characterized fungal and bacterial communities during inoculated fermentations of *Vitis vinifera* cv. Nebbiolo grapes from multiple vineyards, integrating chemical and volatilome data. The results revealed distinct temporal dynamics and region-associated patterns in fungal and bacterial communities, together with associated differences in chemical and volatile profiles. These findings indicate that geographical differentiation of grape-associated microbial communities is maintained throughout inoculated fermentation and is associated with distinct microbial succession patterns and chemical profiles.

**IMPORTANCE:**

Yeast and bacterial population dynamics during alcoholic fermentation play a decisive role in shaping wine characteristics, and the composition of microbial communities present on grapes at harvest is known to influence the fermentation process. In this study, fungal and bacterial community patterns associated with vineyard geography remained detectable throughout standardized inoculated fermentations, despite the dominance of a commercial starter culture. These persistent microbial signatures were associated with differences in microbial succession, chemical composition, and wine volatilome, supporting the concept that regional microbial imprints can remain detectable under commercial winemaking conditions.

## INTRODUCTION

Wine fermentation is a biologically complex process driven by structured microbial successions that strongly influence fermentation kinetics, chemical composition, and sensory attributes of the final product (1, 2). Although *Saccharomyces cerevisiae* usually prevails during alcoholic fermentation, owing to its high fermentative efficiency and stress tolerance, a diverse assemblage of non-*Saccharomyces* yeasts exerts metabolic activity, particularly during the early and intermediate phases of fermentation. These microorganisms contribute to wine differentiation through the modulation of organic acids, glycerol production, and the release of aroma precursors, thereby increasing wine complexity (3–5). Importantly, the composition and succession of fermentative microbiota, encompassing fungi and bacteria, are shaped by environmental and ecological factors rather than being stochastic, suggesting that local microbial consortia may exert a decisive influence on fermentation trajectories and wine typicity (1, 6, 7).

In recent years, the concept of terroir has gained increasing importance, highlighting that microbial assemblages associated with grape surfaces and must are influenced by vineyard location, climatic conditions, soil, plant compartments, viticultural practices, and *S. cerevisiae* genotype (1, 6–12). These communities are not distributed randomly but rather exhibit biogeographical structuring, with fungal communities being more sensitive to local environmental conditions (13, 14). Such spatial differentiation has been associated with variability in fermentation dynamics, organic acid metabolism, volatile compound production, and sensory expression of wines (15, 16). Although the microbial terroir concept has been extensively investigated in widely cultivated international grape varieties, most studies have focused on spontaneous fermentations or vineyard-associated microbiota, leaving the impact of inoculated fermentation on vineyard-specific microbial community patterns largely unexplored (6, 17, 18).

The “Nebbiolo” grape variety is renowned for its demanding viticultural requirements and its enological distinctiveness, yielding wines that are characterized by elevated acidity, pronounced tannins, and remarkable aromatic complexity (19). The preponderance of non-*Saccharomyces* yeasts has been documented, with significant findings of *Hanseniaspora uvarum*, *Starmerella bacillaris*, and *Metschnikowia pulcherrima* during the initial fermentation stages (3, 20). Moreover, lactic acid bacteria such as *Oenococcus oeni* and *Lactobacillus* spp. play an essential role in malolactic fermentation, contributing to the modulation of acidity and the production of aroma-active compounds (21, 22). Nebbiolo is cultivated in various viticultural areas with distinct characteristics, making it an ideal model to investigate whether vineyard-specific microbial communities retain their geographical identity during standardized inoculated fermentations (19).

We hypothesize that geographically distinct Nebbiolo vineyards harbor distinct grape-associated microbial communities and that these geographical differences persist throughout standardized inoculated fermentations despite the dominance of the commercial starter culture. Based on this framework, the objectives of this study were to (i) characterize the chemical composition of musts from 38 Nebbiolo vineyards across three geographically distinct areas; (ii) describe the temporal dynamics of fungal and bacterial communities during fermentation with particular attention to early-stage transitions; (iii) assess whether geographical origin is associated with differences in initial microbial diversity and community composition; (iv) evaluate the persistence of geographical patterns in microbial communities throughout inoculated fermentation; and (v) explore potential associations between microbial profiles, chemical parameters, and wine aroma composition.

By integrating microbial, chemical, and volatilome analyses, we aimed to determine whether geographical variation in grape-associated microbial communities is reflected in the chemical and aromatic profiles of the resulting wines under standardized inoculated fermentation.

## MATERIALS AND METHODS

### Sample collection and weather parameters of the sites

Thirty-eight *Vitis vinifera* L. Nebbiolo cv. grape samples, each from a different vineyard location, were harvested during the period from 2 to 11 October 2023. Each sample consisted of 25 kg of healthy grape berries, collected randomly from different clusters across the vineyard. The vineyards were located in the Piedmont and Lombardy regions (Valtellina) in Italy (Fig. 1; Table S1). Three geographical areas were considered: North Piedmont (NP; 15 vineyards), South Piedmont (SP; 13 vineyards), and Valtellina (VT; 10 vineyards). All vineyards operate under conventional management practices. The three geographic areas were defined *a priori* based on spatial separation among sampling sites and distinct environmental and viticultural conditions. SP is characterized by a mean inter-site distance of ~10–15 km, whereas NP shows a broader spatial distribution (~30–40 km) and is located at an approximate distance of ~80–120 km from SP. VT exhibits a mean inter-site distance of ~10–20 km and is located ~150–200 km from the Piedmont areas. Distances were calculated as straight-line (Euclidean) distances between sampling sites.

**Fig 1:**
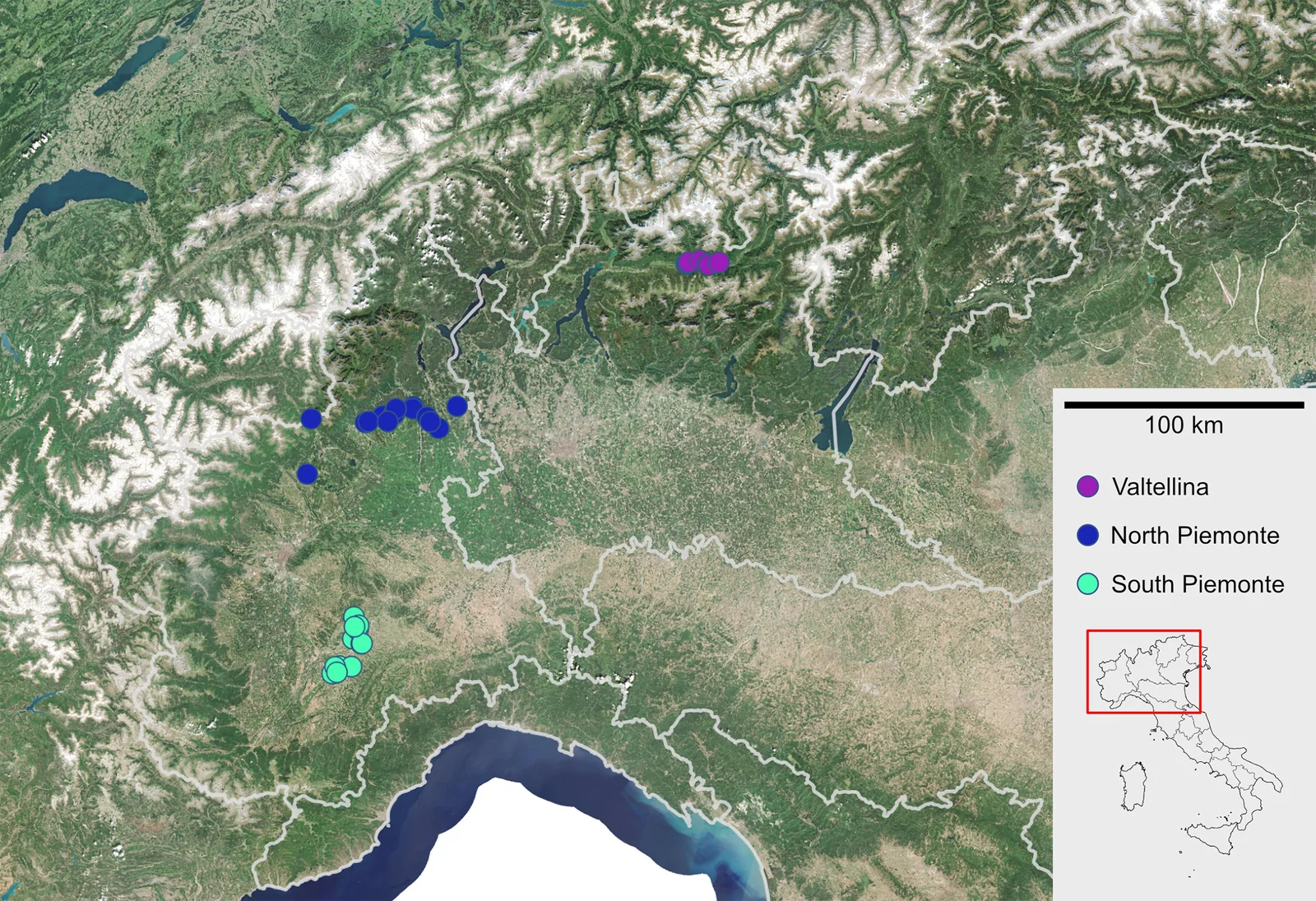
Geographic distribution of the 38 vineyard sampling sites included in this study. Each pin represents a distinct vineyard, grouped into three main areas: North Piedmont (blue, *n* = 10–22; 37–38), South Piedmont (aquamarine, *n* = 01–09; 23–24; 35–36), and Valtellina (deep pink, *n* = 25–34). Detailed information on each sampling site, including coordinates and sampling dates, is provided in Table S1. Contains modified Copernicus Sentinel data 2021 (European Space Agency, Paris, France) for Sentinel data prepared and made available by the Bundesamt für Kartographie und Geodäsie (Frankfurt, Germany).

The climatic data (daily average minimum temperature, daily average maximum temperature, and precipitation) were obtained from ARPA meteorological stations located as close as possible to the sampled vineyards in each province. Specifically, data were retrieved from the following meteorological stations: Biella–Biella (S2566), Cuneo–Alba Tanaro (429), Novara–Momoagogna (442), Turin–Parella Chiusella (S2887), Vercelli–Lozzolo (S2579), and Sondrio (107) (23, 24). In terms of temperature, 2023 was the second-warmest year since 1957. The average annual temperature in Piedmont was around 11.2°C, which is 1.3°C higher than the reference climatic period. Regarding the cumulative annual rainfall in Piedmont, 2023 recorded an average rainfall of 944 mm. Precipitation events were scarce until April, returning to average levels by mid-June (25).

### Fermentation conditions at winery scale

The winemaking process was carried out at the Cantina Sperimentale Bonafous of the University of Turin, located in Chieri (Turin). A total of 38 independent vineyard samples were subjected to fermentation trials and monitored at four time points (days 0, 3, 7, and 14). Each grape sample was destemmed and crushed using a TEMA destemmer–crusher (Enoveneta, Piazzola Sul Brenta, Italy), ensuring that the winery equipment was sanitized with hot water between the processing of different samples (26). The resulting grape mash was transferred to sterile 20 L glass demijohns for fermentation. According to the manufacturer’s instructions, each sample was inoculated with 20 g/hL *S. cerevisiae* Lalvin BRL97 active dry yeast (Lallemand, Montreal, Canada), including 30 g/hL of GoFerm Protect protectant (Lallemand), supplemented with 15 g/hL of nutrients (Fermaid E, Lallemand), and then maceration occurred for 14 days. To minimize the risk of contamination, the demijohns were placed in an area of the winery that had not been previously used for fermentation. Demijohns were equipped with airlocks to prevent contamination while allowing CO_2_ release. The process was carried out at a controlled temperature of 25°C. Two pumping-overs were performed each day using dedicated winery equipment; all materials were cleaned and sanitized before and after each use following standard winery sanitation procedures (including hot water and ethanol-based sanitization). Crushed grape samples and fermenting must samples were collected using sterile serological pipettes and transferred into sterile 15 mL tubes during alcoholic fermentation (on days 0, 3, 7, and 14). Day 0 corresponded to the time before inoculation with *S. cerevisiae*. At each sampling time, three technical subsamples were collected from each fermentation vessel and processed throughout the analytical workflow. The samples were transported to the microbiology laboratory of DISAFA in Alba only for analytical purposes. After fermentation, the grape pomace was manually pressed due to the small scale of the trials. Sulfur dioxide (50 mg/L) was then added, and the wines were bottled and stored at a controlled temperature for 6 months before further analyses.

### Genomic DNA extraction and amplicon sequencing

A volume of 1 mL (in triplicate) of the crushed sample on day 0 and 1 mL (in triplicate) of the fermenting must on days 3, 7, and 14 were centrifuged at 14,000 × *g* for 5 min using an Eppendorf Centrifuge 5430 R (Sigma-Aldrich Co., Milan, Italy). Genomic DNA was extracted from all samples using the QIAcube HT system, an automated high-throughput nucleic acid purification platform in a 96-well format, according to the DNeasy 96 PowerSoil Pro QIAcube HT Kit (Qiagen S.r.l., Milan, Italy). Amplification of the D1 domain of the 26S rRNA encoding gene was performed with the primer pair LS2-MF (5′-GAG TCG AGT TGT TTG GGA AT-3′) and NL4R (5′-GGT CCG TGT TTC AAG ACG G-3′), as described by Mota-Gutierrez et al. (27). For bacterial community profiling, the V3-V4 region of the 16S rRNA gene was amplified using the primers 16S-F (5′-TCG TCG GCA GCG TCA GAT GTG TAT AAG AGA CAG CCT ACG GGN GGC WGC AG-3′) and 16S-R (5′-GTC TCG TGG GCT CGG AGA TGT GTA TAA GAG ACA GGA CTA CHV GGG TAT CTA ATC C-3′) and the protocol established by Klindworth et al. (28). PCR products were purified using the Agencourt AMPure Kit (Beckman Coulter, Milan, Italy), indexed using the Nextera XT Index Kit (Illumina, San Diego, CA, USA) according to the manufacturer’s protocol, and subsequently sequenced on the NovaSeq PE250 platform (Illumina).

### Bioinformatics

Raw sequencing reads were processed using QIIME2 software (version 2024.10) (https://docs.qiime2.org/). Primer sequences were removed using the Cutadapt plugin (29). Paired-end reads were processed in QIIME 2 (30) using the DADA2 plugin for quality filtering, denoising (dada2 denoise-paired with the following parameters: trunc_len_f:242 and trunc_len_r:244), filtering (--p-trunc-q 10), and chimera removal (pooled method), resulting in the generation of amplicon sequence variants (ASVs) (31). Taxonomic assignment of the microbial communities was performed by aligning the ASVs against the SILVA database (version 138.1) for the 16S rRNA gene (32) and a custom database for the 26S rRNA region (27). Taxonomic assignment was performed using the q2-feature-classifier plugin with the VSEARCH consensus classifier (33), using a 100% sequence identity threshold (--p-perc-identity 1). ASVs assigned to mitochondria, chloroplasts, or unassigned taxa were removed before downstream analyses. The resulting table of amplicon sequence variants was subsequently filtered at 0.5% to remove singletons and very rare ASVs. Due to the different taxonomic resolution, bacterial taxa were conservatively reported at the genus level, whereas fungal taxa were assigned at the species level when the identity threshold was met. When this threshold was not achieved, assignments were restricted to the highest supported taxonomic rank.

### Primary chemical metabolites and color analysis of grape must and wine samples

At days 0 (crushing), 3, 7, and 14, all fermentation trials were sampled to investigate their basic composition in terms of sugars (sum of glucose and fructose), malic acid, acetic acid, lactic acid, glycerol, and ethanol. For this aim, the samples were microcentrifuged at 13,000 × *g* for 5 min (5430 centrifuge, Eppendorf, Hamburg, Germany), and the supernatant was filtered with a 0.45 μm PTFE membrane filter into a 2 mL vial. The filtered sample was then analyzed using high-performance liquid chromatography using an Agilent Technologies 1260 system (Santa Clara, CA, USA) following the method of Schneider et al. (34). The operating conditions were 0.7 mL/min isocratic flow of 0.0013 mol/L sulfuric acid solution, Aminex HPX-87H (Bio-Rad, Hercules, CA, USA) column with a Micro-Guard Cation H cartridge (Bio-Rad), all kept at 65°C during analysis, 5 μL injection volume, and detection with a refractive index detector except for malic acid, for which a UV detector set at 210 nm was used. Analyte identification and quantification were performed by means of retention time using calibration curves from pure standards. Data are expressed as concentration (g/L for all analytes, except ethanol, which is expressed as percentage by volume).

On days 3, 7, and 14, the developing wines were also analyzed for their basic color features. For this aim, wines were microcentrifuged at 13,000 × *g* for 5 min (5430 centrifuge, Eppendorf, Hamburg, Germany), and then the spectrum at 360–800 nm wavelength was recorded against deionized water, using 1 mm glass cuvettes, with a UV-1800 spectrophotometer (Shimadzu Corporation, Tokyo, Japan). Color intensity and hue were calculated according to the OIV-MA-AS2-07B method (35) on a 10 mm optical path.

### Free volatile compounds

The bottled wines were analyzed following the method reported by Slaghenaufi and Ugliano (36), with some modifications made to determine the presence of varietal and fermentative compounds. Into a 20 mL vial, 3 g of NaCl was weighed, and then a 5 mL aliquot of wine was added together with 5 mL of ultrapure water. Fifty microliters of internal standard solution (1-heptanol at 60 mg/L in 10% [vol/vol] ethanol) was added. The vial was hermetically sealed with an aluminum screw cap and a silicone septum. The vial was carefully shaken to dissolve the NaCl prior to analysis. The Gerstel MPS 2 autosampler (Gerstel GmbH & Co., Mülheim an der Ruhr, Germany) was used for headspace solid-phase microextraction (HS-SPME) and injection. The sample was equilibrated at 40°C for 10 min. Subsequently, volatile compounds were extracted and adsorbed onto a 50/30 μm divinylbenzene–carbon wide range–polydimethylsiloxane (DVB/C-WR/PDMS) SPME fiber (Agilent Technologies, Santa Clara, CA, USA) at 40°C for 60 min with continuous stirring at 250 rpm. The volatile compounds were then desorbed into the injector port at 250°C for 5 min in splitless mode.

Gas chromatography-mass spectrometry (GC-MS) analysis was carried out using an Agilent 7890A gas chromatograph coupled to an Agilent 5975C quadrupole mass spectrometer. The separation of volatile compounds was performed using a DB-WAX capillary column (30 m × 0.25 mm i.d., 0.25 µm film thickness) from Agilent Technologies, with helium serving as the carrier gas at a flow rate of 1.0 mL/min. The GC oven temperature was programmed as follows: isothermal at 40°C for 5 min, then increased at a rate of 2°C/min to 200°C, which was maintained for 10 min, and subsequently increased at 5°C/min to 220°C, maintaining this temperature for 5 min. The mass spectrometer was operated in the positive electron ionization mode at 70 eV with an ion source temperature of 230°C and a quadrupole temperature of 150°C. All mass spectra were acquired in full scan mode with a mass range of 30–350 *m/z*. GC-MS data were processed using Agilent MSD ChemStation software. The detected volatile compounds were identified by comparing the mass spectra and retention time for each compound with the respective pure standard or the mass spectra reported in the literature and the NIST database ( https://webbook.nist.gov/chemistry/). With regard to quantification, volatile compounds were determined (µg/L) using external calibration with pure standards (Sigma-Aldrich, Milan, Italy) when applicable. Otherwise, results were reported as microgram equivalents of internal standard (1-heptanol) per liter of wine sample.

### Statistical analysis

Statistical analyses and data visualization were performed in R ([37]; RStudio Desktop version 2025.05.1+513, Posit Software, Boston, MA, USA) and Python (3.14.3). Python analyses employed NumPy for scientific computing (38), Pandas for data structuring and manipulation (39), Matplotlib and Plotly for graphical visualization (40), Scikit-learn for machine learning implementation (PCA and SparsePCA [Sparse principal component analysis]) (41), and Scikit-bio for bioinformatic purposes (CLR transformation) (42). Raw count data were converted to relative abundances for downstream analyses and visualization. Samples with extremely low sequencing depth (<1,000 reads) were excluded from downstream analyses. All retained fungal samples showed Good’s coverage values >0.98, whereas all retained bacterial samples showed Good’s coverage values >0.95. Due to the compositional nature and high sparsity of such microbiological data, a tailor-made chemometric pipeline was then employed. Features with relative abundances >0.5% in at least five samples were retained. Following feature selection, data were subjected to closure to restore compositional constraints, zeros were replaced using multiplicative replacement (43), and CLR transformation was performed (44). This chemometric workflow refers to the subsequent application of principal component analysis (PCA) and its sparse variant. PCA was initially used to identify potential outliers in the bacterial and fungal taxonomic data sets, both globally and within each geographical area. Outliers were identified using score distance (*T*^2^) and the orthogonal distance (*Q*) diagnostic statistics, evaluated through influence plots at a customized confidence level (1 − *α*) (45). PCA was subsequently used to explore overall patterns in microbial community structure and chemical parameters throughout the fermentation process. To better account for the high sparsity of the microbiome data sets, SparsePCA was additionally performed, while conventional PCA was retained as a benchmark for comparison (46). Alpha diversity was estimated as ASV richness using the QIIME 2 observed_features metric after rarefaction to 1,000 reads per sample (30). Differences among geographical groups were assessed using the Kruskal–Wallis test, followed by Benjamini–Hochberg-adjusted pairwise Wilcoxon rank-sum tests. Beta diversity was evaluated using Bray–Curtis dissimilarities and visualized by PCA. Differences in community composition were tested by permutational multivariate analysis of variance (PERMANOVA; 999 unrestricted permutations) using models to evaluate (i) geographical differences at day 0 and (ii) the additive effects of geographical area and fermentation stage (47). Core fungal taxa were identified for each area–time combination using the core_members function of the phyloseq package and merged across groups. Their temporal dynamics were visualized as heatmaps of mean relative abundance, whereas overall abundance patterns were represented using row-wise *Z*-score-standardized heatmaps with hierarchical clustering based on Euclidean distance and Ward’s linkage. Chemical parameters during fermentation were analyzed using linear models fitted separately for each compound, with area as a fixed effect. Model residuals were inspected and met normality assumptions. Estimated marginal means were computed using the emmeans package (48), and pairwise comparisons among areas were performed using Tukey-adjusted contrasts. *P*-values were adjusted using Tukey’s HSD, and confidence intervals were corrected using the Šidák method (49). Results are reported as estimated marginal means ± standard deviation. Relationships between core microbial taxa and chemical/environmental parameters were assessed using Spearman’s rank correlation. Relative abundance data were CLR-transformed after addition of a pseudocount of one to zero values, and correlation coefficients together with their associated *P*-values were visualized as correlation heatmaps (44). Associations between fungal taxa and volatile organic compounds (VOCs) were evaluated by ordinating VOC profiles using principal coordinates analysis (PCoA) based on Bray–Curtis dissimilarities. Microbiological fitting was performed using the envfit function in the vegan package with 999 permutations (47). Only significant vectors (*P* < 0.05) were retained and visualized to highlight VOCs associated with variation in community structure across samples. The analysis was conducted on subsets of samples selected based on extreme values of target yeast presence.

## RESULTS

### Dynamics of microbial communities in Nebbiolo grapes and musts

A total of 152 samples originating from 38 different cv. Nebbiolo vineyards and corresponding inoculated fermentations (four time points in total) were collected. For each sample, three technical subsamples were collected and processed throughout the analytical workflow. The samples were represented by crushed grapes (day 0, before *S. cerevisiae* inoculation) and fermenting must during the progression of alcoholic fermentation (days 3, 7, and 14). A total of 18,659,648 16S rRNA sequences and 60,046,100 26S rRNA sequences were generated from these samples and resolved into 451 bacterial and 290 fungal ASVs. Taxonomic classification was based on sequence matches showing 100% identity to the reference database. For fungal communities, taxonomic assignment was achieved at the species level for a proportion of ASVs, whereas for bacterial communities, confident classification was generally limited to the genus level. To investigate whether region-associated fungal and bacterial community patterns remained detectable throughout inoculated fermentation, a PCA and a SparsePCA were conducted. In both the fungal and bacterial cases, (Fig. 2A and B, respectively), the best results were achieved by using an iterative strategy for alpha (sparsity-controlling parameter) hyperparameter optimization: various alpha values were tested to minimize the difference between the variance explained with canonical PCA and that explained by SparsePCA. The score plot from SparsePCA agrees with the results obtained by using traditional PCA. This means that the Sparse variant of PCA can account for a similar amount of variance to that explained by classic PCA: in both cases, a difference of no more than 10% between variances was observed. The trends of the fungal and bacterial communities were similar. For fungal communities in crushed grapes (day 0), the samples were mostly distributed in the lower left quadrant of the graph, which was characterized by negative values along both first and second components. During the initial fermentation phase (day 3), almost all the samples gradually started to move along the positive value of PC1. From the intermediate time to the end of fermentation (days 7 and 14), the samples shifted toward more positive values along PC2, highlighting how the time factor is well described by both “sparse” principal components. For the bacterial communities, a similar trend, but in the opposite direction, can be observed. Core fungal and bacterial taxa were detected throughout the fermentation process. They were defined as those occurring in at least 50% of the samples with a minimum detection threshold of 0.1% relative abundance. Figure S1 shows the log10-transformed relative abundance distributions of the eight fungal species and eight bacterial genera defined as core taxa. *S. cerevisiae* was the dominant fungal species, showing the highest abundance values overall, followed by *Starm. bacillaris* and *Aureobasidium proteae*, which showed intermediate levels and broad variability among samples. Bacterial communities displayed a less skewed distribution than fungi, with a more even representation across taxa. *Lactobacillus sensu lato* was the most abundant group, followed by *Streptococcus* and *Clostridium*, all showing intermediate abundances and moderate variability, while the remaining genera occurred at lower levels and were more heterogeneous across samples.

**Fig 2:**
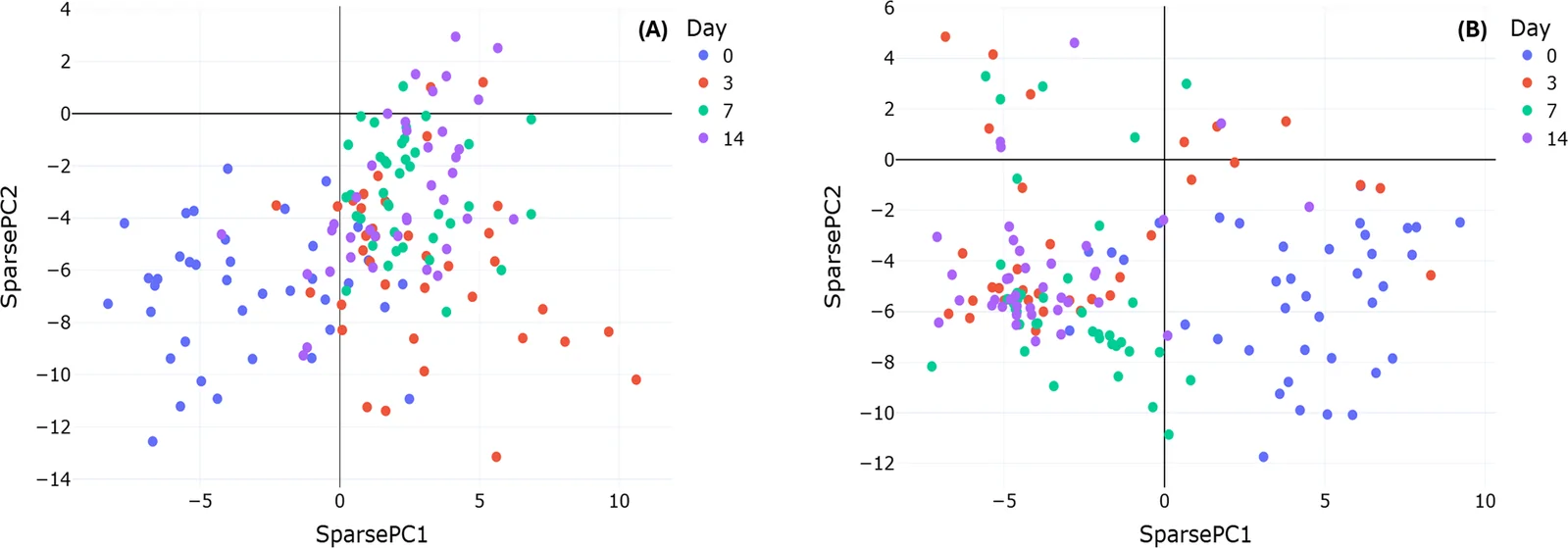
Sparse principal component analysis score plots illustrating the temporal dynamics of fungal (**A**) and bacterial (**B**) community compositions during Nebbiolo grape fermentation. Each point represents the mean of three replicates analyzed for each vineyard collected at one of four time points: T0 (crushed grapes), T1 (start of fermentation), T2 (mid-fermentation), and T3 (end of fermentation). Sampling times are coded according to different colors.

### Variation in microbial communities of crushed Nebbiolo grapes across vineyards

To evaluate geographical differences in the indigenous grape-associated microbiota independently of the commercial starter culture, all analyses in this section were performed exclusively on crushed grape samples collected before *S. cerevisiae* inoculation (day 0). To identify potentially atypical samples, Hotelling’s *T*^2^- and *Q*-statistics were calculated following traditional PCA analysis, since the Sparse version of PCA was unable to describe suitable values of explained variance. The resulting *T*²-*Q* influence plots at a 90% confidence level and relative to the crushed grapes (day 0) were obtained separately for the three geographical areas and for fungi (shown in Fig. S2) and bacteria. Only one sample slightly exceeded the dotted orange line representing the critical value for *Q* (03-CN) in the plot representing the fungal taxonomic data set of South Piedmont samples (Fig. S2a). Similarly, only one sample (15-BI) exhibited a *Q*-value slightly above the 90% critical value (Fig. S2b) from North Piedmont samples and one (25-SO) for Valtellina (Fig. S2c). Within the bacterial taxonomic data set, not reported in Fig. S2, just two samples, 9-CN and 32-SO, showed *Q*-values slightly above the *Q*-critical limit. These results indicated the absence of major outliers within each geographical area; therefore, all samples were retained for subsequent analyses and grouped into three macro-areas: South Piedmont, North Piedmont, and Valtellina. Alpha diversity measurements were used to illustrate variation in the ASV richness of fungal and bacterial species across crushed grape samples from the three different geographical areas (Fig. 3). No significant differences in fungal ASV richness were detected among geographical areas following the Kruskal–Wallis test and pairwise Wilcoxon rank-sum tests with Benjamini–Hochberg correction for multiple comparisons (Wilcoxon, *P* > 0.05). In contrast, bacterial ASV richness differed significantly between the VT area and both SP (*P* < 0.05) and NP (*P* < 0.001), whereas no significant difference was observed between SP and NP (*P* > 0.05).

**Fig 3:**
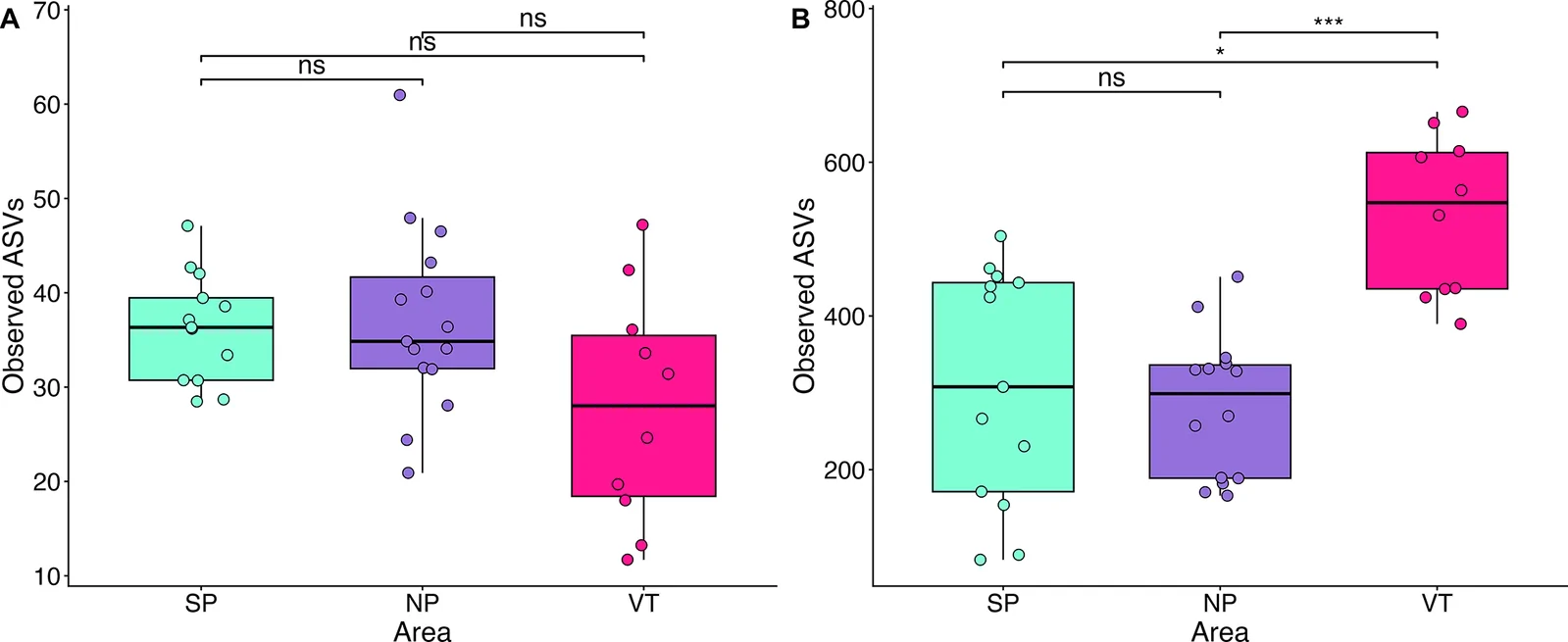
Fungal (**A**) and bacterial (**B**) ASV richness in crushed grape samples collected before inoculation (day 0) across NP, SP, and VT. Boxes represent the distribution of observed ASV richness, with central lines indicating median values. Differences among geographical areas were assessed using the Kruskal–Wallis test followed by pairwise Wilcoxon rank-sum tests with Benjamini–Hochberg correction. Asterisks indicate adjusted significance levels (*P < 0.05; ***P < 0.001); ns, not significant.

To determine whether microbial community composition differed among the three geographical areas, beta-diversity was assessed using Bray–Curtis dissimilarity, and statistical significance was evaluated by PERMANOVA. Significant differences were detected for both fungal (*R*^²^ = 0.197, *P* = 0.001) and bacterial communities (*R*^²^ = 0.197, *P* = 0.003), indicating that geographical origin explained approximately 20% of the total variation in microbial community composition. To visualize these statistically supported differences, PCA was performed on the crushed grape (day 0) taxonomic data sets (Fig. S3). This time filter was able to eliminate both the time-dependent contribution and the potential effect associated with the evolution of artificial inoculum. The pre-processing steps before PCA are the same as described in “Statistical analysis,” above. The PCA showed clustering of samples according to geographical origin, consistent with the PERMANOVA results. Taxa present at a relative abundance of at least 0.5% in five or more samples were selected for further analysis. Amplicon sequencing showed that the fungal profile of crushed grapes was dominated by different filamentous fungi like *A. proteae*, *Botrytis cinerea*, *Cladosporium* spp., and different yeasts, including *Hanseniaspora uvarum*, *S. cerevisiae*, and *Starm. bacillaris* in all the areas (Fig. S4). *A. proteae* was the most prevalent species in the SP area, with a relative frequency of 25.0%, followed by *Starm. bacillaris* at 18.6% and *S. cerevisiae* at 13.8%. In contrast, the VT and NP areas showed comparable species distributions, with relatively small differences in the frequencies of the dominant species (*Starm. bacillaris*, 41.7% in VT and 28.5% in NP; *A. proteae*, 18.3% in VT and 26% in NP; and *H. uvarum*, 12.6% in VT and 8.6% in NP). The bacterial communities consisted mainly of the genera *Gluconobacter*, *Komagataeibacter*, *Lactobacillus sensu lato,* and *Streptococcus. Gluconobacter* was the most prevalent genus in the SP area, with a relative frequency of 32.0%, followed by *Bacillus* with a relative frequency of 7.6% and *Lactobacillus sensu lato* at 6.9%. In contrast, the VT and NP areas showed comparable distributions, as they shared the same dominant genera with relatively limited differences in their relative abundances. *Komagataeibacter* was the most prevalent genus in both areas (32% for VT and 11.8% for NP). The second- and third-most abundant genera were *Gluconobacter* and *Lactobacillus sensu lato*, although in reversed order between the two areas (*Gluconobacter*, 14.8% in NP and 10.6% in VT; *Lactobacillus sensu lato*, 4.8% in NP and 10.6% in VT).

### Temporal dynamics and persistence of region-associated microbial communities during inoculated fermentation

Analysis of the microbiota composition during alcoholic fermentation revealed significant variations associated with sampling time and, consequently, fermentation progression, as well as with geographical area. Permutational analysis of variance of fungal composition confirmed that both factors significantly contributed to explaining variation in fungal communities (*P* < 0.001), collectively explaining 73.5% of the observed variability in Bray–Curtis distance between samples. Hierarchical aggregation of yeast taxa revealed coherent abundance patterns that were consistently shaped by both time and geographical origin (Fig. 4A). At the early stage, prior to the inoculum (day 0), the NP area showed a particularly strong positive correlation with several taxa, including *Lachancea thermotolerans*, *Pichia californica*, and *Zygosaccharomyces bailii*. This trend was attenuated in later stages (from day 3 to day 14). A similar profile was observed in SP, with *Z*-score peaks for *Hanseniaspora* spp.*, Alternaria* spp.*, Cladosporium* spp., *Metschnikowia fructicola aff.,* and *Aspergillus niger* decreasing over advancing time. A distinct composition characterized the VT area, with fewer species having a relatively high correlation with the early stages, such as *Debaryomyces hansenii*, *Pichia kluyveri,* and *Starm. bacillaris*. A few species showed higher relative abundance in the second stage (day 3): *Pseudozyma tsukubaensis* for NP, *Wickerhamiella pararugosa* and *Wickerhamiella azyma* for SP, and *Candida tropicalis* and *Diutina rugosa* for VT. *S. cerevisiae* exhibited an even more delayed pattern in all areas, showing a particularly low abundance in the early stage, which then increased in the later stages. These patterns were confirmed by the analysis of indicator species, conducted for each area using the IndVal approach. This analysis identified 22 species significantly associated (*P* < 0.05) with at least one of the three areas. In the NP area, *Hanseniaspora osmophila* (*P* = 0.001), *Kazachstania servazzii* (*P* = 0.009), *Kluyveromyces dobzhanskii* (*P* = 0.004), *Starmerella lactis-condensi* (*P* = 0.003), and *Lachancea quebecensis* (*P* = 0.008) stood out. The SP area, instead, showed an indicator profile dominated by *Rhodosporidiobolus odoratus* (*P* = 0.005), *Cladosporium tassiana* (*P* = 0.003), *Alternaria alternata* (*P* = 0.009), *Pringsheimia smilacis* (*P* = 0.003), and *Ustilago cynodontis* (*P* = 0.015). In VT, *Hanseniaspora occidentalis* (*P* = 0.004), *Zygotorulaspora* spp. (*P* = 0.003), and *Clavispora asparagi* (*P* = 0.017) were significantly associated. The NP + SP combination presented associations with *Sporobolomyces roseus* (*P* = 0.015), *Cladosporium variabilis* (*P* = 0.027), *Pichia occidentalis* (*P* = 0.028), *Penicillium citrinum* (*P* = 0.028), *Lachancea lanzarotensis* (*P* = 0.039), *Trichosporon asahii* (*P* = 0.037), and *Bullera albus* (*P* = 0.027). A significant association also emerged between the NP and VT areas, represented by the species *Hanseniaspora vineae* (*P* = 0.006) and *Penicillium* spp. (*P* = 0.049).

**Fig 4:**
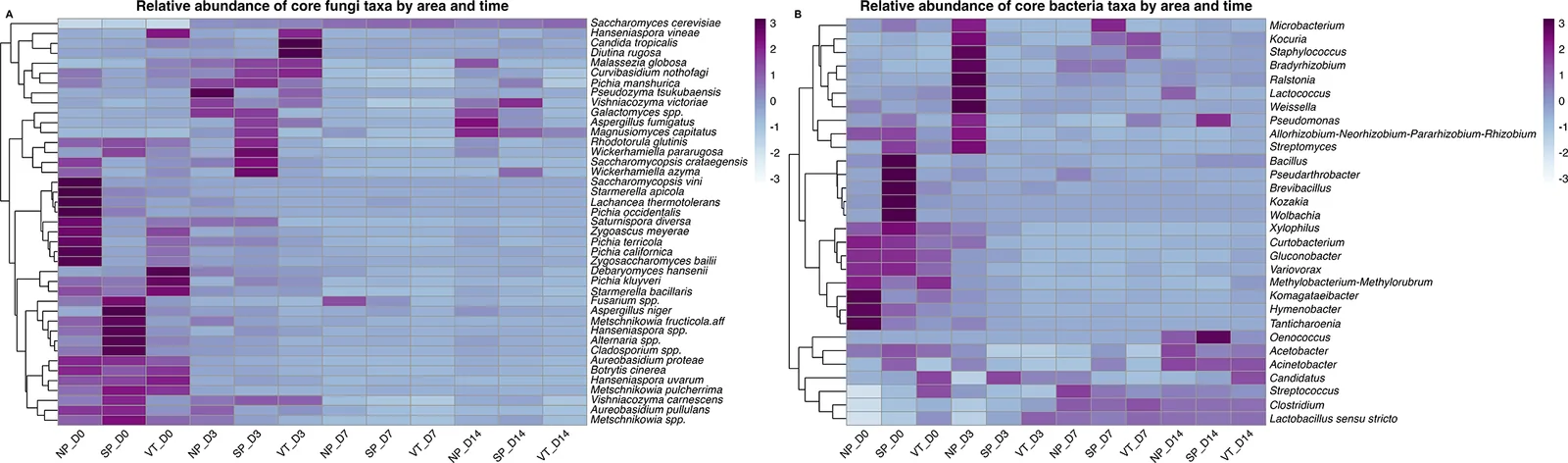
Heatmap showing the scaled relative abundances (*Z*-score) of core fungal (**A**) and bacterial (**B**) taxa across samples grouped by area and fermentation time. The *X*-axis represents the combinations of geographical area and sampling time (e.g., NP_D0 = North Piedmont at day 0), while the *Y*-axis lists the fungal and bacterial taxa identified as core in at least one group (present in ≥50% of samples with a relative abundance ≥1%). Both taxa and sample groups are hierarchically clustered based on abundance profiles to visualize patterns of community composition, temporal dynamics, and spatial differences across the fermentation process.

As observed for fungal communities, bacterial community composition during alcoholic fermentation was significantly influenced by both sampling time and geographical area (Fig. 4B). PERMANOVA confirmed the significant effects of both factors (*P* < 0.001), which together explained 29.4% of the variation in Bray–Curtis dissimilarity among samples. Among the most represented taxa, *Oenococcus* was particularly present in the final phase of fermentation (day 14), especially in samples from SP and NP. Similar trends were observed for *Acetobacter* and *Acinetobacter*, which were more abundant in the final phase of fermentation (day 14), especially in samples from NP. In this area, a high abundance of *Komagataeibacter*, *Gluconobacter*, *Methylobacterium-Methylorubrum*, *Hymenobacter,* and *Tanticharoenia* was observed on the crushed grapes (day 0), showing an early peak in abundance that progressively reduced in the subsequent fermentation phases, with the bacteria almost completely disappearing by day 14. Several bacterial genera were present at higher than average concentrations at day 3 and day 7, such as *Staphylococcus*, *Lactococcus*, *Pseudomonas*, *Allorhizobium-Neorhizobium-Pararhizobium-Rhizobium*, and *Bradyrhizobium*. The SP had a different distribution since most of the bacteria had their relative abundance peak at time zero and then decreased in the subsequent phases. The *Microbacterium* genus also differed from the others, peaking in the intermediate phases (days 3 and 7). In contrast, VT had a more homogeneous bacterial distribution throughout the fermentation process with fewer significant peaks in abundance. Bacterial analysis of indicator species, conducted using the IndVal approach for each area, identified 26 genera significantly associated (*P* < 0.05) with at least one of the three areas. In the NP area, the species *Pluralibacter* (*P* = 0.031) and *Aeromonas* (*P* = 0.038) were significantly associated. The SP group was characterized by *Skermanella* (*P* = 0.002), *Exiguobacterium* (*P* = 0.014), *Gluconacetobacter* (*P* = 0.035), *Granulicatella* (*P* = 0.019), *Planomicrobium* (*P* = 0.016), *Tumebacillus* (*P* = 0.034), and *Clavibacter* (*P* = 0.045). The VT group also showed clear associations with *Micromonospora* (*P* = 0.017), *Coriobacterium* (*P* = 0.001), *Alcaligenaceae* (*P* = 0.007), *Azospirillum* (*P* = 0.010), *Kingella* (*P* = 0.016), *Hungatella* (*P* = 0.037), and *Geobacillus* (*P* = 0.043). Regarding the group combinations, combining NP and VT highlighted three significantly associated species, with *Paenibacillus* showing the highest association value (stat = 0.550, *P* = 0.017). Finally, the association between SP and VT was the richest in terms of indicator species, with seven taxa detected. Among these, *Acetitomaculum* and *Brevibacillus* showed the highest association values (stat = 0.568 and 0.487, respectively), both accompanied by particularly marked levels of statistical significance (*P* = 0.007).

### Chemical parameters and correlation with microbial ecology

The evolution of the chemical parameters from grape to wine is presented in Table 1. Basic physicochemical parameters of the grapes/musts are reported in Table 1 and Table S2 for acidity parameters before the inoculum. These latter showed significant differences among the three geographic areas, with VT exhibiting higher total acidity and lower pH than NP and SP. No stuck fermentations were observed, and most fermentations were completed by day 7. Three fermentations of grapes from the SP area (06-CN, 24-CN, and 36-CN) were slower, finishing by day 14. Due to these three fermentations, the mean total sugar content has a rather high standard deviation. Significant differences were observed among the various metabolites at all stages in the three analyzed regions. Regarding SP and VT, meaningful differences emerged in the total sugar levels, with the SP region demonstrating higher sugar content in both crushed grapes and at the end of fermentation (235.66 g/L for SP and 216.42 g/L for VT at day 0 and 0.30 g/L for SP and 0.02 g/L for VT at day 14). Significant differences in acetic acid content were observed between SP and both VT and NP, with SP demonstrating significantly higher levels (0.06 g/L for NP, 0.11 g/L for SP, and 0.07 g/L for VT at day 3; 0.07 g/L for NP, 0.13 g/L for SP, and 0.07 g/L for VT at day 7; and 0.10 g/L for NP, 0.17 g/L for SP, and 0.11 g/L for VT at day 14). The opposite trend was observed for malic acid, showing a higher concentration in VT and NP from crushed grapes until the end of the fermentation process (2.18 g/L for NP, 1.03 g/L for SP, and 2.45 g/L for VT at day 0; 3.09 g/L for NP, 1.60 g/L for SP, and 3.06 g/L for VT at day 3; 2.99 g/L for NP, 1.76 g/L for SP, and 3.14 g/L for VT at day 7; and 2.57 g/L for NP, 1.55 g/L for SP, and 3.03 g/L for VT at day 14). Significant differences in lactic acid concentrations were observed between NP and VT during fermentation (0.22 g/L for NP and 0.15 g/L for VT at day 3; 0.20 g/L for NP and 0.14 g/L for VT at day 7), but these leveled off in the final wine. The lactic acid concentrations at the end of fermentation differed among the three areas, but these differences were not significant due to the high standard deviation. This was due to the presence of two vineyards in NP (1.39 g/L for 17-BI and 1.65 g/L for 18-BI) and one in SP (1.06 g/L for 06 CN), which had notably higher than average concentrations. During fermentation, there were no significant differences in glycerol and ethanol. They only appeared at the end of fermentation in the SP and VT areas in terms of ethanol production, with the SP area showing a higher percentage than the VT and NP areas (12.07% [vol/vol] for NP, 13.52% [vol/vol] for SP, and 12.53% [vol/vol] for VT at day 14). Regarding color, only a significant difference was observed in terms of color intensity at the end of fermentation between SP and VT, with significantly higher values for SP (4.01 for SP and 3.10 for VT).

**TABLE 1:** Standard chemical parameters measured at different stages of the fermentation process from grape to wine^*a*^

Parameter	Sampling time (days)	Area	EMmean ± SD	Significance
Sugars (sum of glucose and fructose, g/L)	0	NP	215.96 ± 16.89	a
		SP	235.66 ± 11.51	b
		VT	216.42 ± 8.79	a
	3	NP	41.29 ± 32.76	a
		SP	81.73 ± 47.38	b
		VT	55.17 ± 24.68	ab
	7	NP	0.32 ± 0.15	a
		SP	9.06 ± 18.59	a
		VT	0.15 ± 0.16	a
	14	NP	0.14 ± 0.15	ab
		SP	0.30 ± 0.31	b
		VT	0.02 ± 0.06	a
Acetic acid (g/L)	3	NP	0.06 ± 0.03	a
		SP	0.11 ± 0.05	b
		VT	0.07 ± 0.04	a
	7	NP	0.07 ± 0.03	a
		SP	0.13 ± 0.06	b
		VT	0.07 ± 0.04	a
	14	NP	0.10 ± 0.04	a
		SP	0.17 ± 0.07	b
		VT	0.11 ± 0.05	a
Malic acid (g/L)	0	NP	2.18 ± 0.58	b
		SP	1.03 ± 0.56	a
		VT	2.45 ± 0.73	b
	3	NP	3.09 ± 0.55	b
		SP	1.60 ± 0.35	a
		VT	3.06 ± 0.92	b
	7	NP	2.99 ± 0.44	b
		SP	1.76 ± 0.35	a
		VT	3.14 ± 0.76	b
	14	NP	2.57 ± 0.61	b
		SP	1.55 ± 0.48	a
		VT	3.03 ± 0.73	b
Lactic acid (g/L)	3	NP	0.22 ± 0.07	b
		SP	0.17 ± 0.08	ab
		VT	0.15 ± 0.03	a
	7	NP	0.20 ± 0.04	b
		SP	0.15 ± 0.04	a
		VT	0.14 ± 0.01	a
	14	NP	0.40 ± 0.48	a
		SP	0.23 ± 0.25	a
		VT	0.07 ± 0.01	a
Glycerol (g/L)	3	NP	9.20 ± 0.92	a
		SP	8.69 ± 1.46	a
		VT	8.99 ± 0.63	a
	7	NP	10.14 ± 0.72	a
		SP	10.32 ± 0.67	a
		VT	10.09 ± 0.56	a
	14	NP	10.04 ± 0.70	a
		SP	10.35 ± 0.58	a
		VT	9.99 ± 0.52	a
Ethanol (% [vol/vol])	3	NP	9.52 ± 1.42	a
		SP	8.70 ± 2.11	a
		VT	9.28 ± 0.99	a
	7	NP	11.97 ± 0.67	a
		SP	12.88 ± 0.97	b
		VT	12.37 ± 0.65	ab
	14	NP	12.07 ± 0.67	a
		SP	13.52 ± 0.91	b
		VT	12.53 ± 0.58	a
Color intensity (A.U.)	3	NP	3.68 ± 0.74	a
		SP	4.84 ± 1.34	b
		VT	3.97 ± 0.91	ab
	7	NP	3.92 ± 0.59	a
		SP	4.81 ± 1.65	a
		VT	3.69 ± 0.86	a
	14	NP	3.39 ± 0.50	ab
		SP	4.01 ± 1.18	b
		VT	3.10 ± 0.53	a
Hue	3	NP	0.65 ± 0.07	a
		SP	0.62 ± 0.07	a
		VT	0.62 ± 0.06	a
	7	NP	0.74 ± 0.08	a
		SP	0.71 ± 0.11	a
		VT	0.72 ± 0.07	a
	14	NP	0.86 ± 0.10	a
		SP	0.84 ± 0.12	a
		VT	0.87 ± 0.09	a

^*a*^ Sampling time 0 refers to crushed grapes before inoculation; sampling times 3, 7, and 14 indicate days of fermentation. Data are expressed as estimated marginal means (EMmean) ± standard deviation. Within each sampling time, different letters indicate statistically significant differences among geographical areas (letters increase with mean values; the lowest value corresponds to “a”) (Tukey-HSD test, *P *< 0.05). Although all parameters were analyzed at each sampling time, results at time 0 are reported only for selected compounds, as some parameters are less informative prior to fermentation.

To statistically assess the effects of fermentation time, geographical origin, and their interaction on the overall chemical composition, a PERMANOVA was performed. The full model was significant (*R*^²^ = 0.61, *P* = 0.001), explaining a substantial portion of the variance in chemical composition. When examining individual effects, fermentation time was the main driver of compositional variation (*R*^²^ = 0.598, *P* = 0.001), whereas geographical origin was not significant (*R*^²^ = 0.0066, *P* = 0.608). The interaction between time and origin was also not significant (*R*^²^ = 0.0025, *P* = 0.626), indicating that temporal dynamics were consistent across vineyard areas. Principal components analysis revealed that the first principal component reflects the fermentation progression (PC1 = 63.0%), with early-stage samples (day 0, crushed grapes) separated from the fermentative stages (days 3, 7, and 14) (Fig. 5). This shift is driven by a significant decrease in sugar (glucose and fructose) concentrations, alongside increasing levels of ethanol, glycerol, and lactic acid, in line with alcoholic fermentation progressing. Although the confidence ellipses representing the three vineyard areas partially overlap, discernible spatial patterns were observed. Examining the fermentation times more closely revealed that there was already a significant difference in the chemical parameters between the three geographical areas (*R*^²^ = 0.60, *P* = 0.001) at time zero (Fig. S5). Samples from the VT region showed higher levels of organic acids and glycerol, while NP and SP samples were characterized by higher sugar content.

**Fig 5:**
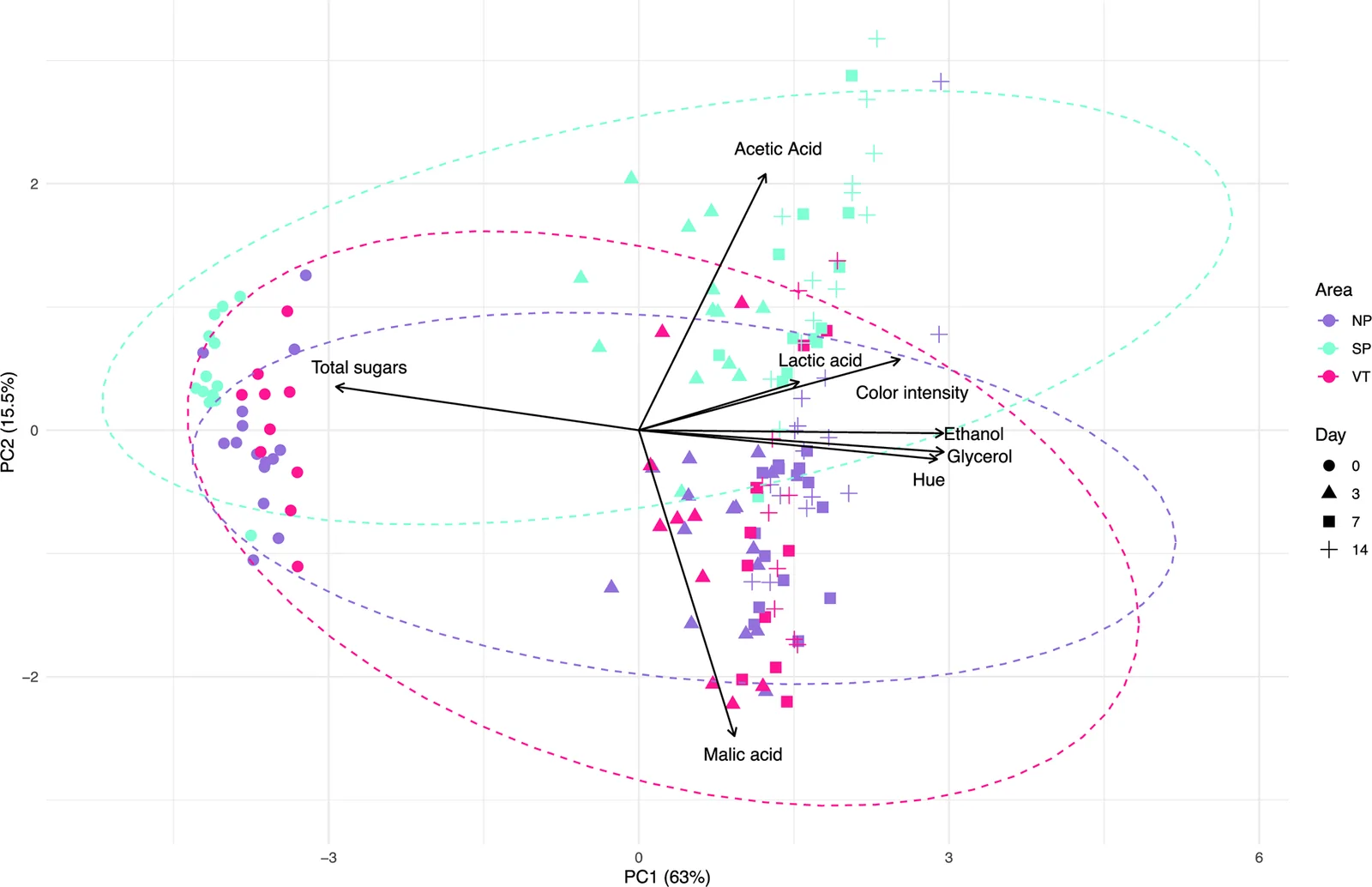
PCA showing chemical parameters throughout the winemaking process, from intact grapes to the final stages of alcoholic fermentation. Each shape represents a sampling time. Sampling time 0 refers to crushed grapes before inoculation; sampling times 3, 7, and 14 indicate days of fermentation. Each color represents a geographical origin (NP, North Piedmont; SP, South Piedmont; and VT, Valtellina).

Figure S6 shows the correlations between the main climatic and chemical parameters and microorganisms that were present at a relative abundance of at least 0.5% in five or more samples collected at the time of grape crushing (day 0). The analysis was carried out separately for fungal and bacterial communities in each geographical area. In the NP area, significant correlations were observed between fungal taxa and environmental or chemical parameters. *B. cinerea* showed negative correlations with altitude (*P* = 0.024) and total acidity (*P* = 0.040), while *Aureobasidium pullulans* was positively associated with glucose (*P* = 0.033) and total sugars (*P* = 0.037). *Starm. bacillaris* was negatively correlated with altitude (*P* = 0.045), and a positive association was observed for *Zygoascus meyerae* (*P* = 0.025). Among bacteria, *Methylobacterium-Methylorubrum* showed a significant positive correlation with temperature (*P* = 0.044). The SP area displayed the highest number of significant associations, particularly among fungi. *Cladosporium* spp. and *Alternaria* spp. showed positive correlations with total sugar (*P* = 0.034 and *P* = 0.036) and glucose (*P* = 0.002 and *P* = 0.0004), while *Alternaria* spp. also showed a positive correlation with fructose (*P* = 0.008). *S. cerevisiae* was negatively correlated with total sugars, glucose, and fructose (*P* = 0.007, *P* = 0.019, and *P* = 0.002). *A. proteae* was positively associated with altitude (*P* = 0.003), whereas *A. pullulans* and *B. cinerea* were negatively correlated with temperature and rainfall, respectively (*P* = 0.010 and *P* = 0.038). *Starm. bacillaris* and *H. uvarum* showed negative correlations with relative humidity (*P* = 0.015 and *P* = 0.027). Among bacteria, *Curtobacterium* was negatively correlated with altitude (*P* = 0.044), while *Streptococcus* showed a negative correlation with temperature (*P* = 0.035) and a positive one with altitude (*P* = 0.044). *Staphylococcus* was positively associated with rainfall (*P* = 0.028). In the VT area, fungal correlations included positive associations of *S. cerevisiae* with temperature (*P* = 0.043), total sugars (*P* = 0.015), glucose (*P* = 0.011), and fructose (*P* = 0.021), and negative correlations with relative humidity (*P* = 0.043) and rainfall (*P* = 0.043). *Cladosporium* spp. was negatively associated with altitude (*P* = 0.016) and *Alternaria* spp. with total acidity (*P* = 0.031). Bacterial communities showed more numerous associations. *Lactobacillus sensu lato* and *Corynebacterium* were positively correlated with rainfall and relative humidity and negatively with temperature (all *P* = 0.005). In contrast, *Methylobacterium-Methylorubrum* showed the opposite trend, with negative correlations with rainfall and relative humidity and a positive correlation with temperature (all *P* = 0.005). Finally, *Gluconobacter* and *Terrisporobacter* exhibited opposite correlations with pH, being positively (*P* = 0.039) and negatively (*P* = 0.009) associated, respectively. A comparative analysis highlighted that the number and direction of significant correlations varied across the three geographical areas.

### Geographical patterns in wine volatilome and associated microbial communities

Volatile compounds were determined by GC/MS analysis 6 months after the end of the fermentation period. Nebbiolo grape fermentation resulted in the detection of 39 compounds (Table S3). To investigate the potential effect of the geographical origin of the grapes on the volatile compounds, a PCA was conducted on the 38 wine samples analyzed. To obtain a two-dimensional plot in which “three-dimensional” information is condensed, an initial PCA, in which the first three PCs were selected, was followed by a factorial analysis applying the Varimax algorithm (orthogonal rotation) on the loadings matrix. Then, the new score matrix was computed by multiplying the original data matrix with the new rotated loadings matrix. Finally, a new PCA was applied on the new score matrix, selecting only the first two components. The resulting score plot is shown in Fig. 6. This plot revealed a clear separation between SP and NP samples, as can be noted along the bisector between the first and third quadrants. As might be expected, VT samples show a small intra-class variability because the sampling area is more limited and quite homogeneous with respect to SP and NP areas.

**Fig 6:**
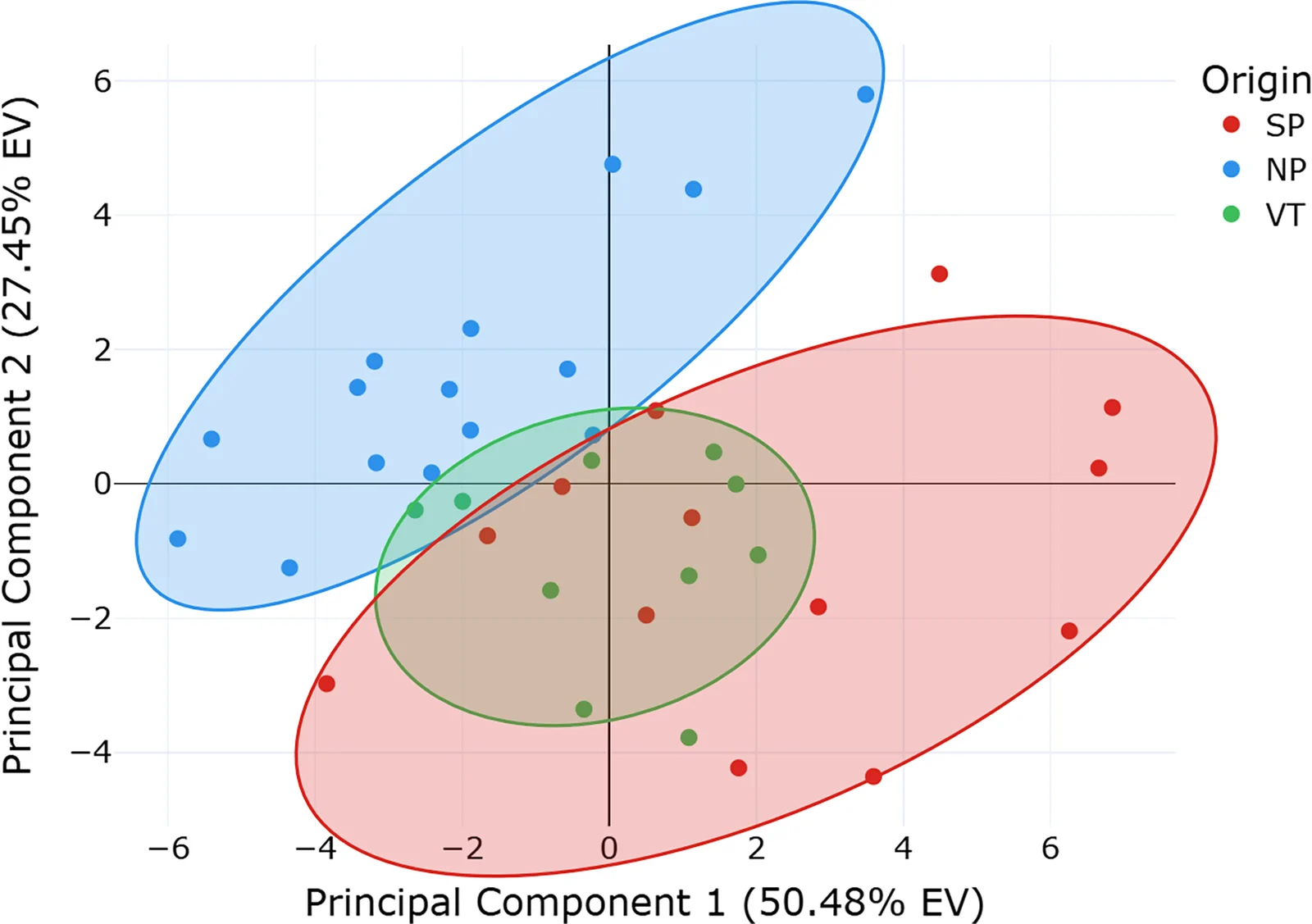
Principal component analysis score plots—after Varimax rotation—illustrating the composition of volatile compounds 6 months after the end of Nebbiolo grape fermentation. Each point represents a sample, and each color represents a geographical area: red, SP; blue, NP; and green, VT.

As shown in Fig. 3, differences in fungal and bacterial richness (*P* < 0.05) were observed between the SP and VT areas. For this reason, only these two areas were considered when exploring whether the richness of the grape microbial community can influence the composition of the final product’s volatile compounds (wine, 6 months after the end of alcoholic fermentation). Using the Bray–Curtis dissimilarity index and performing a PERMANOVA test, a statistically significant separation was confirmed (*P* < 0.05, *R*^²^ = 0.251). A principal coordinates analysis of volatile compounds revealed a clear separation between the samples from the SP and VT areas. The first two coordinates explained a significant proportion of the total variance (PCoA1 = 79.60%, PCoA2 = 8.86%) by distributing the samples according to their geographical origin.

In order to explore possible associations between microbial communities and aroma profiles, envfit analysis was applied using the relative abundances of fungi (Fig. 7A) and bacteria (Fig. 7B) of greatest oenological interest and VOC concentrations as explanatory variables for the positioning of samples in ordinal space. The vectors represented the taxa and volatile compounds that were significantly correlated with the sample distribution along the first two axes of the PCoA (*P* < 0.05). The VOCs were grouped into five chemical classes, namely higher alcohols, volatile fatty acids, esters, terpenes, and norisoprenoids. Among the volatile compounds, ethyl esters exhibited variable correlations with the principal components. Some compounds, such as ethyl acetate, ethyl butanoate, ethyl decanoate, and ethyl dodecanoate, were negatively correlated with PC1 and positively correlated with PC2, whereas others, such as ethyl heptanoate and ethyl octanoate, showed different directions of association, highlighting that esters are not uniformly distributed along a single gradient. Alcohols and terpenes were associated with negative values of PC2, with 2-phenylethanol and isobutanol showing the strongest correlations. Fungal species were distributed in a similar way to volatile compounds. Fungal species largely mirrored the patterns of VOCs: *A. alternata*, *Cladosporium* spp.*,* and *Alternaria* spp. were positively associated with ethyl esters, while *A. niger* and *Torulaspora delbrueckii* were linked to alcohols and terpenes. Among bacteria, only the genera *Acetobacter*, *Pseudomonas*, and *Staphylococcus* were statistically significant in the envfit analysis, all positively associated with alcohols and terpenes, with *Staphylococcus* exhibiting the strongest correlation.

**Fig 7:**
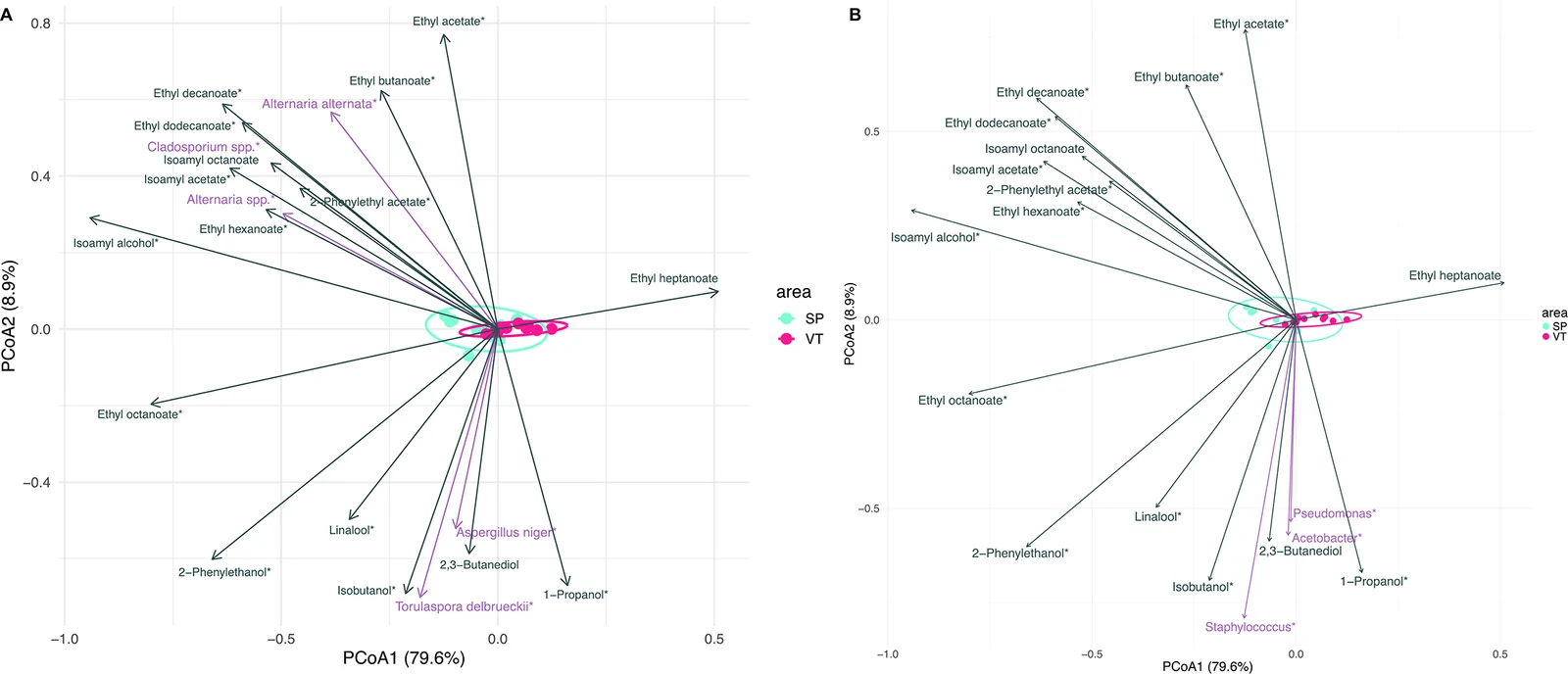
Principal coordinates analysis plot based on dissimilarity (Bray–Curtis distance; *P* < 0.05) among volatile compounds collected from two geographical areas: SP and VT. Each point represents a sample, and ellipses denote the distribution and dispersion within each geographical group. Arrows represent the significant variables contributing to sample separation: volatile compounds (*P* < 0.05) and the most relevant fungal species (**A;** *P* < 0.05); volatile compounds and the most relevant bacterial genera (**B**; *P* < 0.05). The direction of each arrow reflects the axis of greatest variance for that variable, while the length indicates the strength of its contribution to the ordination. The PERMANOVA test confirmed a significant separation between areas (*R*^2^ = 0.251, *P* < 0.05).

## DISCUSSION

The microbial communities involved in wine fermentation are characterized by high levels of diversity and dynamism and consist of a complex network of yeasts, bacteria, and fungi that interact throughout the winemaking process, thereby significantly influencing wine quality and typicity (50). Previous studies on Nebbiolo have shown that vineyard-associated non-*Saccharomyces* yeasts contribute to fermentation ecology and that environmental factors influence grape microbial communities (51, 52). However, under inoculated fermentations, the contribution of indigenous microbiota may be partially masked by the dominance of the commercial *S. cerevisiae* starter culture.

In this study, microbial diversity was high during the early fermentation stages but declined progressively as *S. cerevisiae* became dominant, a pattern consistent with the use of a starter culture. *S. cerevisiae* is well established for its dominance in alcoholic fermentation and its contributions to microbial stability and sensory complexity (53). While the inoculation predictably drove the dominance of *S. cerevisiae*, several non-*Saccharomyces* taxa remained detectable throughout fermentation, indicating that part of the original vineyard-associated community structure was retained despite inoculation. In addition to *S. cerevisiae*, the presence of *Starm. bacillaris* and *A. proteae* was observed to be consistent across all samples, thereby suggesting a stable fungal core community. *Starm. bacillaris* has been frequently observed to be active in early fermentation, contributing to glycerol production and aromatic complexity (4). In contrast, *A. proteae*, though rarely reported in winemaking, belongs to a genus with considerable enzymatic versatility and adaptability to must conditions (54). *Metschnikowia* spp., *M. pulcherrima*, *A. proteae*, *B. cinerea,* and *H. uvarum* consistently appeared as stable grape-associated populations characterized by comparable abundances across SP, NP, and VT. This reflects low regional specialization and high ecological ubiquity, suggesting that these taxa are consistently associated with grape surfaces across geographical areas (1, 15). Nevertheless, confirmation of a stable core microbiota will require validation across multiple vintages and production contexts. In contrast, bacterial communities exhibited a more even distribution and less temporal structuring, with genera such as *Lactobacillus sensu lato*, *Gluconobacter*, and *Komagataeibacter* appearing at different stages. *Lactobacillus sensu lato* was found to be particularly abundant during early fermentation, potentially contributing to sugar consumption and acid modulation (21). Acetic acid bacteria such as *Gluconobacter* and *Komagataeibacter* were detected at different stages of winemaking. Despite the capacity of these taxa to oxidize sugars and ethanol and their potential contribution to volatile acidity and aroma modulation (55), their detection within the present study is based on DNA profiling and does not provide direct evidence of metabolic activity. The presence of *Streptococcus*, which is not typical of grape microbiota, may reflect vineyard or phyllosphere origin, potentially contributing to microbial interactions during fermentation (56).

The microbial profile of crushed grapes revealed distinctive spatial differentiation among the three areas considered in this study. Alpha diversity analyses showed reduced fungal richness in VT compared to SP and NP, although these differences were not statistically significant. In contrast, bacterial ASV richness was significantly higher in VT than in SP and NP. These results suggest that geographical origin may be associated with differences in microbial composition and in the balance between fungal and bacterial communities at the pre-fermentative stage (57). Beta diversity analyses confirmed a stronger biogeographical signal in fungi than in bacteria, consistent with earlier reports indicating that fungal communities are more sensitive to viticultural and climatic conditions (13, 14, 58). The species distributions suggested patterns consistent with differential prevalence across environmental contexts. *Starm. bacillaris* was more prevalent in NP and VT, areas characterized by cooler climatic conditions (data not shown), in agreement with previous observations linking this species to lower-temperature environments (3, 59). In contrast, *A. proteae*, *A. niger*, and *M. fructicola* were enriched in the SP region, in line with reports describing their frequent occurrence in warmer viticultural areas (54, 60, 61). Osmotolerant species like *P. californica*, *P. terricola*, *Z. bailii,* and *Z. meyerae* were common in NP. While these species are known for their tolerance to osmotic stress, their regional prevalence should be interpreted cautiously, as no direct environmental measurements supporting this association were available (50, 62). Bacterial genera demonstrated increased overlap between regions, although some distinctions were observed: *Bacillus* and *Brevibacillus* were more frequent in SP, known for their ecological association with warmer and drier vineyard environments, and their frequent association with soil and plant surfaces. Conversely, *Komagataeibacter* and *Tanticharoenia* were more dominant in NP, consistent with their reported association with cooler and more humid environments and soil-derived contamination (6). *Corynebacterium* was particularly prevalent in the VT area, possibly reflecting local soil characteristics and vineyard management practices (14). Overall, these findings indicate that grape-associated microbial communities were geographically differentiated before inoculation, providing the baseline for evaluating their persistence during fermentation.

Geographical origin and sampling time explained a larger fraction of variation in fungal communities (73.5%) than in bacterial ones (29.4%), further supporting the higher spatial resolution of fungal signatures (6, 7). Importantly, fungal community differences remained detectable despite inoculation with the commercial *S. cerevisiae* starter culture, indicating that inoculation did not completely erase the initial geographical structuring of fungal communities. The presence of a clear microbial succession over time, with environmental species dominating the early stages and the progressive dominance of *S. cerevisiae* in the final stages, is a well-established pattern (53). NP fermentation samples were found to be enriched in yeasts such as *L. thermotolerans, P. californica,* and *Z. bailii*, which are known for their ability to modulate acidity and tolerate stress (3, 62). In SP, early-stage species included *A. niger*, *Hanseniaspora* spp*., Alternaria* spp*.,* and *Cladosporium* spp., which are typically associated with soils and plant surfaces (5, 63–65). The presence of *M. fructicola* (*aff.*), a species adapted to high-sugar and low-acidity conditions on grape surfaces, is consistent with this ecological context (66). The VT area showed distinct positive correlations with *P. kluyveri, D. hansenii,* and *Starm. bacillaris*, taxa linked to cold-resistant environments and aromatic complexity (4, 62, 67). Late-stage fermentations in all areas featured *Oenococcus,* consistent with its central role in malolactic fermentation (68). *Acetobacter* was also detected at this stage; however, its presence should be interpreted with caution, as DNA-based profiling does not provide evidence of metabolic activity. Previous studies have reported the persistence of acetic acid bacteria during late fermentation or post-fermentative stages, whose presence as residual or inactive populations is limited by ethanol concentration, low oxygen availability, and sulfur dioxide (6, 69, 70). Indicator species analysis further confirmed spatial microbial signatures. In the context of fungal taxonomy, the SP class was found to be enriched in filamentous fungi and plant-associated yeast species such as *C. tassiana*, *A. alternata*, and *U. cynodontis*. In contrast, NP was characterized by rare or underexplored taxa such as *K. servazzii* and *S. lactis-condensi* (71–73), while NP and VT shared *H. vineae* and *Penicillium* spp., indicating overlap in cooler zones rather than convergence (74). Taken together, these observations support the hypothesis that the initial biogeographical organization of the microbiota is only partially reshaped by inoculation and remains associated with local environmental conditions, rather than demonstrating a direct causal role of indigenous microbiota in determining fermentation outcomes (6, 75).

The chemical profiles of fermenting musts provided context for these microbial patterns. The higher acetic acid level observed in SP may be consistent with a higher abundance of acetic acid bacteria such as *Gluconacetobacter* and *Skermanella*, likely reflecting rapid sugar oxidation in warm climates (55). In NP, the increased persistence of malic acid during fermentation is consistent with the lower relative abundance of lactic acid bacteria such as *Lactobacillus sensu lato* and *Oenococcus,* indicating limited malolactic activity (21). In the SP area, the prevalence of *A. proteae* and *Cladosporium* spp. coincided with faster sugar conversion and higher ethanol content, which may reflect differences in must composition and fermentation dynamics rather than direct microbial causation (54). The occurrence of variable species distribution across sites likely reflects ecological plasticity (6, 13). Microbial communities at grape crushing (day 0) showed region-specific correlations with environmental and chemical parameters, highlighting the role of microbial terroir. Overall, SP showed stronger links between fungal taxa and variables such as sugar composition, altitude, and rainfall, whereas VT was characterized by more pronounced bacterial associations, particularly with temperature, relative humidity, and pH. NP displayed fewer but still significant relationships, mainly involving fungi and temperature- or nutrient-related variables. Several expected microbe–metabolite associations were not strongly detected, likely reflecting the multifactorial and dynamic nature of fermentation ecosystems, in which microbial abundance does not necessarily correspond directly to metabolic activity or metabolite accumulation at a given sampling stage (76). Overall, these findings suggest that geographical variation in microbial community composition is associated with distinct chemical trajectories during fermentation.

Six months after fermentation, the volatile composition of the analyzed wines reflected the varietal typicity of Nebbiolo, while also displaying clear geographically distinct characteristics, especially between SP and VT. These composition differences were associated with variation in the initial microbial richness, indicating that differences in the initial grape microbiota were reflected in the volatile composition of the resulting wines. In SP, volatile compounds were more strongly associated with long-chain esters and fusel alcohols. Ethyl octanoate is commonly produced during rapid fermentations, dominated by *S. cerevisiae* and high nitrogen availability. The presence of this ester is associated with richer, riper fruit notes but can also reflect increased fermentative vigor and nutrient assimilation. In this area, fungal species such as *A. alternata*, *Cladosporium* spp.*,* and *Alternaria* spp. were more prevalent and co-occurred with higher levels of ethyl esters, possibly by enhancing precursor release or modulating yeast activity (77, 78). The presence of indicator species such as *Gluconacetobacter* in SP corresponds to the higher levels of acetic acid detected, consistent with the genus’s known ability to oxidize ethanol and increase volatile acidity. While moderate levels of volatile acidity can contribute positively to wine complexity, excessive levels proved to have a detrimental effect on sensory quality (55). Conversely, in VT, ethyl heptanoate was more abundant and associated with *P. kluyveri, D. hansenii,* and *H. uvarum*, suggesting a potential contribution of these taxa to ester-related profiles under cooler vineyard conditions. However, previous studies have shown that *D. hansenii* can reduce overall ester production during fermentation, likely as a consequence of its high esterase activity or limited precursor availability (79)*. Starm. bacillaris*, *H. uvarum,* and *H. vineae* were reported to be linked to the production of medium-chain ethyl esters and terpenes, thereby contributing to greater floral and varietal complexity (4, 16, 80). *T. delbrueckii* contributed to a reduction in acetaldehyde and acetic acid levels, supporting its known roles in osmotic tolerance and aromatic refinement (78). Bacterial genera, including *Pseudomonas* and *Staphylococcus*, occurred with higher levels of higher alcohols and terpenic compounds, suggesting possible indirect or ecological associations. The presence of *Staphylococcus* in SP also necessitates a re-evaluation of its potential enological relevance (81, 82). Collectively, these findings indicate that part of the initial geographical differentiation of grape-associated microbial communities is retained throughout standardized inoculated fermentation and is associated with differences in microbial succession, chemical composition, and wine volatile profiles. Although these associations are consistent with the concept of microbial terroir, they should not be interpreted as evidence of a direct causal role of indigenous microbiota in determining the final wine chemical composition under inoculated fermentation conditions.

### Conclusion

These results indicate that geographically distinct Nebbiolo vineyards harbor differentiated grape-associated microbial communities. Despite inoculation with a commercial starter culture, part of this initial biogeographical structure was retained throughout fermentation. Fungal populations, particularly non-*Saccharomyces* yeasts, exhibited a discernible biogeographical pattern that was associated with environmental conditions and local climate. This spatial signal was detectable at the grape level and partially persisted throughout fermentation despite the dominance of the inoculated starter culture. In contrast, bacterial communities showed weaker geographical structuring but retained functional diversity, with multiple genera exhibiting site-specific patterns that remained detectable during fermentation. Climatic and environmental conditions were associated with the composition of grape microbiota, with cooler conditions favoring cold-adapted fermentative taxa and warmer environments promoting thermotolerant and soil-derived microorganisms. These initial microbial assemblages may therefore reflect region-specific ecological filtering processes acting at the vineyard level. Although correlations were observed between microbial taxa and volatile compounds, including esters, higher alcohols, and terpenes, these relationships should be interpreted as associations between region-associated microbial signatures and wine volatile composition rather than evidence of a direct causal effect of indigenous microbiota under inoculated fermentation conditions. Collectively, these findings demonstrate that standardized inoculated fermentations do not completely obscure regional microbial signatures and support the concept of microbial terroir as a detectable ecological imprint that can persist under commercial winemaking conditions. However, as this study was conducted over a single vintage, further multi-year analyses will be necessary to validate the persistence of these patterns, assess the stability of a core fermentative microbiota, and distinguish site-specific effects from annual variability. A better understanding of the persistence of vineyard-associated microbial signatures under inoculated fermentation may provide new opportunities to improve wine traceability and to better integrate regional microbial diversity into future winemaking strategies.

## Data Availability

The sequencing data set supporting the conclusions of this study is available in the NCBI Sequence Read Archive (SRA) under accession number PRJNA1390442.

## SUPPLEMENTAL MATERIAL

Supplemental figures aem.01510-26-s0001.docx Fig. S1 to S6.

Supplemental tables aem.01510-26-s0002.xlsx Tables S1 to S3.

ASM does not own the copyrights to Supplemental Material that may be linked to, or accessed through, an article. The authors have granted ASM a non-exclusive, world-wide license to publish the Supplemental Material files. Please contact the corresponding author directly for reuse.
