# Supplementary material for: Region-associated microbial signatures persist during inoculated fermentation of Nebbiolo grapes
Source: Appl Environ Microbiol. 2026 Sep 9;92(10):e01510-26. doi: 10.1128/aem.01510-26 (PMC13689010; doi:10.1128/aem.01510-26)
Supplement: Supplementary file 1 [file aem.01510-26-s0001.docx]

# **Supplementary materials**

**Figure S1.** Box-and-whisker plots showing the log10-transformed relative abundance of core taxa identified in fungal (A) and bacterial (B) communities across samples. Core taxa were defined based on prevalence and detection thresholds (detection ≥ 0.1, prevalence ≥ 0.5). Each point represents an individual sample and is colored according to the sampling area (NP in purple, SP in aquamarine and VT in pink), while boxplots indicate the median, interquartile range, and overall distribution of values for each taxon. Median values are highlighted as central points within each distribution. Taxa are ordered by decreasing median log10 relative abundance.

**Figure S2.** Analysis of potential outliers in the fungal taxonomic datasets (T0) from the three geographical areas using Hotelling’s T² vs. Q-residuals model.

**Figure S3.** PCA scores plot showing the geographical-related clusters at t0 for both fungal (A) and bacterial (B) taxonomic datasets. Each color represents a geographical area: red = SP, blue = NP, and green = VT.

**Figure S4.** Relative abundance (%) of fungal (A) and bacterial (B) taxa identified in crushed grape samples (day 0) from three geographical areas: North Piedmont (NP), South Piedmont (SP), and Valtellina (VT). The barplot displays the average community composition, with each taxon shown as a distinct color.

**Figure S5.** PCA showing chemical parameters at the time of crushing (T0). Each color represents a geographical origin (NP = North Piedmont, SP = South Piedmont, and VT = Valtellina).

**Figure S6.** Correlation matrix based on Spearman's rank correlation. Data were transformed using centered log-ratio (CLR) transformation prior to analysis. The image shows the correlations between core fungal (A, C, E) and bacterial (B, D, F) taxa and the main environmental and chemical parameters across the three areas: NP (A, B), SP (C, D), VT (E, F). The X-axis represents the fungal and bacterial taxa with a relative abundance greater than or equal to 0.5% in at least five samples at the moment of crushing (T0), while the Y-axis lists the main environmental and chemical parameters. Correlations were assessed using two-tailed Spearman tests, and statistically significant associations (p < 0.05) are indicated by asterisks (*).

**Figure S1.**

A) B)

**Figure S2.**

**Figure S3.**

**Figure S4.**

A) B)

**Figure S5.**

**Figure S6.**
